# Pharmacoeconomic Analysis of Osimertinib Compared to Earlier‐Generation EGFR Inhibitors in EGFR‐Mutated NSCLC: A Systematic Review

**DOI:** 10.1002/cam4.72129

**Published:** 2026-07-22

**Authors:** Nicola Perrotta, Adriana Coluccia, Francesca Rossi, Giulia Amato, Giacomo Polito

**Affiliations:** ^1^ Policlinico Umberto I Hospital Sapienza University Rome Italy; ^2^ Department of Chemistry and Technology of Drugs Sapienza University Rome Italy

**Keywords:** cost‐effectiveness analysis, EGFR‐mutated non‐small cell lung cancer, health technology assessment, incremental cost‐effectiveness ratio (ICER), osimertinib

## Abstract

**Background:**

Osimertinib has reshaped the treatment of EGFR‐mutated non‐small cell lung cancer (NSCLC), but its high cost raises persistent questions about value for money.

**Objectives:**

To review economic evaluations of osimertinib in EGFR‐mutated NSCLC and identify the drivers of cost‐effectiveness across first‐line, second‐line/sequential, and adjuvant settings in North America and Europe.

**Methods:**

This review was registered with PROSPERO (CRD420251139947) and conducted in accordance with PRISMA 2020. We searched PubMed/MEDLINE, Embase, Web of Science Core Collection, and the Cochrane Library through August 2025 for peer‐reviewed economic evaluations comparing osimertinib with chemotherapy or earlier‐generation EGFR tyrosine kinase inhibitors from a payer perspective. Reporting quality was appraised with CHEERS 2022. Given heterogeneity in models, pricing, and survival extrapolation, results were synthesized narratively, each incremental cost‐effectiveness ratio (ICER) interpreted against its own jurisdiction's willingness‐to‐pay (WTP) threshold.

**Results:**

Nine studies published between 2018 and 2024 were included. First‐line osimertinib improved outcomes but at ICERs of $151,922–$231,123/QALY (US) and €128,343–€273,895/QALY (Europe), exceeding local thresholds. Second‐line T790M‐positive estimates were favorable and context‐dependent ($159,126/QALY US; £41,705/QALY UK under end‐of‐life criteria). Adjuvant results were most divergent: cost‐effective under cure‐fraction assumptions but unfavorable when benefit was modeled as delayed recurrence. Price, overall‐survival (OS) extrapolation, sequencing, and local WTP thresholds were dominant.

**Conclusions:**

Osimertinib confers meaningful clinical benefit, but its value for money is context‐specific and often uncertain at current list prices. These conclusions rest on few heterogeneous models and warrant caution; sustainable use will depend on price reductions, patient prioritization, maturation of OS data, and outcome‐based reimbursement.

## 1 Introduction

Non‐small cell lung cancer (NSCLC) carrying activating epidermal growth factor receptor (EGFR) mutations is a distinct molecular subset that has benefited substantially from tyrosine kinase inhibitors (TKIs). First‐ and second‐generation EGFR TKIs, including erlotinib, gefitinib, afatinib, and dacomitinib, were shown to significantly prolong progression‐free survival (PFS) compared with platinum‐based chemotherapy in advanced EGFR‐mutated NSCLC [1, 2, 3, 4]. However, acquired resistance develops in most patients, most commonly through the T790M gatekeeper mutation, which arises in roughly half of those treated with earlier‐generation TKIs [5, 6]. Osimertinib, a third‐generation irreversible EGFR TKI, was specifically designed to target both sensitizing EGFR mutations and the T790M resistance mutation [7]. In the pivotal FLAURA trial, first‐line osimertinib nearly doubled median PFS compared with first‐generation TKIs (18.9 vs. 10.2 months) [8] and improved median overall survival (OS), from 31.8 months with erlotinib or gefitinib to 38.6 months [9]. In the phase III AURA3 trial, conducted in patients who developed T790M‐mediated resistance after a first‐line EGFR TKI, osimertinib significantly prolonged PFS (10.1 vs. 4.4 months; HR 0.30) and achieved a higher response rate (71% vs. 31%) than platinum‐pemetrexed chemotherapy [7]. Although the final AURA3 OS analysis did not reach statistical significance, largely because of extensive crossover from the chemotherapy arm, the superior disease control established osimertinib as the standard second‐line option after earlier‐TKI failure [10].

These clinical gains come at a substantial price. In the United States, osimertinib costs approximately $17,000 per month at wholesale acquisition cost, which has raised concerns about cost‐effectiveness, particularly when the drug is used for prolonged periods as first‐line or adjuvant therapy [11, 12]. Health technology assessment (HTA) agencies generally require robust economic evidence before reimbursing high‐cost therapies, and since osimertinib entered practice, a number of cost‐utility and budget‐impact analyses have examined its value across first‐line, second‐line or sequential, and adjuvant settings [11, 13, 14]. Several early analyses reported ICERs above conventional WTP thresholds at prevailing prices [11, 15, 16, 17]. A 2018 U.S. analysis based on FLAURA trial reported an ICER of $226,527 per quality‐adjusted life year (QALY) for osimertinib compared with erlotinib, well above the commonly cited US thresholds of $100,000–$150,000 per QALY [11] and European analyses reported ICERs of €273,895 per QALY in Spain and €128,343 per QALY in the Netherlands for first‐line use [13, 14, 15]. These findings have fueled an ongoing debate about whether the incremental benefit of osimertinib justifies its added cost relative to earlier‐generation EGFR TKIs in Western health systems.

This systematic review synthesizes the published economic evidence on osimertinib in EGFR‐mutated NSCLC, focusing on analyses conducted from the perspective of high‐income North American and European health systems across first‐line, second‐line, or sequential, and adjuvant settings. We examine reported incremental costs, QALYs, ICERs, and budget‐impact estimates, interpret each against the WTP threshold applied in its own jurisdiction, and identify the principal drivers of cost‐effectiveness. We also highlight key sources of uncertainty, notably the maturity of OS data in the adjuvant setting, and consider the implications for payers and policymakers seeking to balance innovation with affordability. We frame our conclusions cautiously, in recognition of the small number and heterogeneity of the available studies.

## 2 Materials and Methods

### 2.1 Protocol, Registration, and Reporting

**FIGURE 1:**
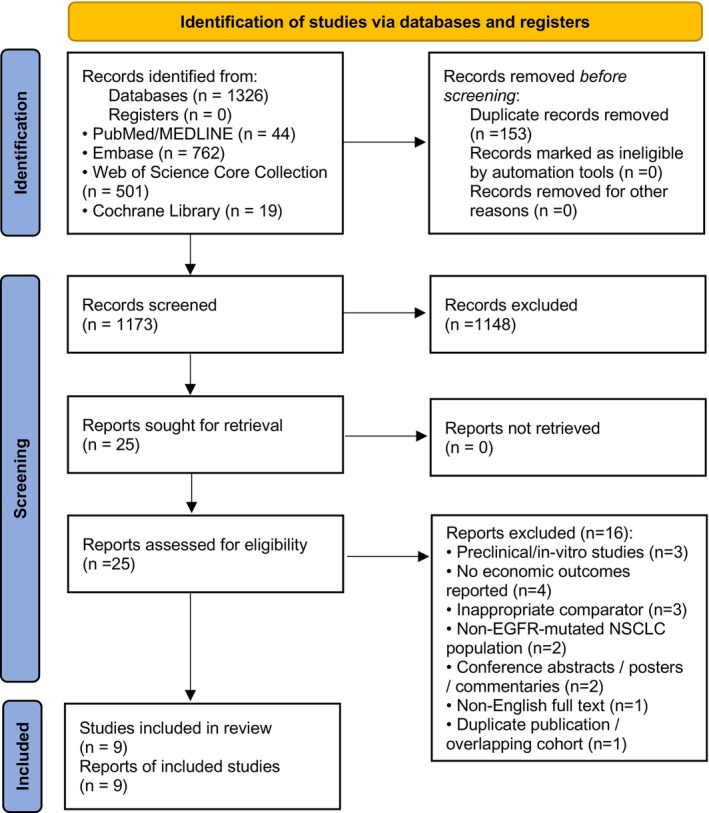
PRISMA 2020 flow diagram of study identification and selection. PRISMA 2020 flow diagram of study identification and selection. A total of 1326 records were identified through database searching (PubMed/MEDLINE, 44; Embase, 762; Web of Science, 501; Cochrane Library, 19) together with supplementary citation tracking. After 153 duplicate records were removed, 1173 records were screened by title and abstract and 25 full‐text articles were assessed for eligibility. Nine economic evaluations met the inclusion criteria and were retained for the narrative synthesis.

The review was prospectively registered with the International Prospective Register of Systematic Reviews (PROSPERO; registration CRD420251139947) before screening was completed, and it was conducted and reported in accordance with the Preferred Reporting Items for Systematic Reviews and Meta‐Analyses (PRISMA) 2020 statement [16]. The PRISMA 2020 flow of records is shown in Figure 1, and a completed PRISMA checklist is provided in the Supplementary Appendix.

### 2.2 Search Strategy and Information Sources

We searched PubMed/MEDLINE, Embase, Web of Science Core Collection, and the Cochrane Library from database inception to 31 August 2025. The strategy combined controlled vocabulary and free‐text terms for the intervention (“osimertinib” or “Tagrisso”), the disease (“non‐small cell lung cancer,” “NSCLC,” “lung adenocarcinoma,” and EGFR‐related terms including “T790M”), and economic outcomes (“cost‐effectiveness,” “cost‐utility,” “economic evaluation,” “pharmacoeconomics,” “ICER,” “QALY,” or “budget impact”). To capture sequential‐treatment analyses, earlier‐generation EGFR TKIs (gefitinib, erlotinib, afatinib, dacomitinib) and chemotherapy were also combined with the economic terms. The complete database‐specific Boolean strings are reported verbatim in the Supplementary Appendix to allow full replication. Searches were restricted to English‐language, peer‐reviewed studies in human subjects. We additionally screened the reference lists of all eligible articles (backward citation searching) and used Google Scholar for forward citation tracking. Conference abstracts, editorials, letters, narrative reviews, preprints, and gray literature or HTA documents lacking complete peer‐reviewed methodological detail were not eligible; the rationale for, and consequences of, these decisions are discussed explicitly as limitations (Section 4).

### 2.3 Eligibility Criteria and Study Selection

After de‐duplication, two investigators independently screened titles and abstracts. Full‐text reports were assessed against pre‐specified criteria: (1) adult patients with confirmed EGFR‐mutated advanced or resected NSCLC; (2) full economic evaluations, budget‐impact analyses, or model‐based pharmacoeconomic studies comparing osimertinib with platinum‐based chemotherapy, first‐ or second‐generation EGFR TKIs, placebo/observation, or sequencing strategies; (3) first‐line, second‐line/T790M‐positive, sequential, or adjuvant use of osimertinib; (4) reporting of economic outcomes such as incremental costs, QALYs, ICERs, WTP thresholds, sensitivity analyses, or budget impact; and (5) a payer, health‐system, or clearly relevant societal perspective situated in North America or Europe. Studies were excluded if they lacked original pharmacoeconomic outcomes, used an inappropriate comparator, focused on non‐EGFR‐mutated or non‐NSCLC populations, were not available as full peer‐reviewed articles, or duplicated another included analysis. Two analyses reported results for both a Western and a non‐Western jurisdiction (United States and China) [17, 18]; these were included on the basis of their North American analyses, and their Chinese results are reported only as descriptive context, not as part of the Western evidence base. Disagreements at the title/abstract or full‐text stage were resolved by discussion between the two reviewers; where consensus could not be reached, a third investigator adjudicated. The geographic restriction was applied a priori to improve comparability across reimbursement frameworks, drug pricing structures, and WTP thresholds, and is treated throughout as a limitation rather than as evidence of global generalizability.

### 2.4 Data Extraction

Two reviewers independently extracted data using a standardized form, and any discrepancies were resolved by a third reviewer [16]. Extracted variables included publication details (authors, year, country), clinical setting and population, modeling approach (Markov, partitioned‐survival or state‐transition), analytic perspective, time horizon and discount rate, interventions and comparators, key clinical inputs and survival‐extrapolation methods, cost inputs (drug acquisition, administration, monitoring, adverse‐event management), utility values and their sources, base‐case outcomes (incremental costs, QALYs, ICERs), the WTP threshold used by the authors, and the authors' conclusions regarding cost‐effectiveness. We also extracted the results of one‐way and probabilistic sensitivity analyses (PSAs), cost‐effectiveness acceptability curves (CEACs), and scenario analyses (such as price reductions or alternative survival assumptions). All costs were extracted and are reported in the original currency of each publication. We deliberately did not convert currencies or adjust for inflation or purchasing power parity, because each model was built for a jurisdiction‐specific decision context and embedded local prices, discounts, reimbursement rules, and thresholds; converting these interdependent inputs would distort the original analyses. To enable meaningful cross‐study comparison without conversion, each ICER is interpreted relative to the WTP threshold of its own jurisdiction (i.e., as a multiple of, or distance from, the local threshold) rather than in absolute monetary terms. The absence of currency normalization is acknowledged as a methodological limitation in Section 4.

### 2.5 Quality Assessment

Reporting quality was appraised using the Consolidated Health Economic Evaluation Reporting Standards (CHEERS) 2022 checklist [19]. We recorded compliance with key items, including the description of the model structure, justification of the time horizon and perspective, transparency of costing methods, and presentation of sensitivity analyses. In parallel, we qualitatively examined potential sources of bias in model inputs and assumptions, supported by the robvis visualization tool [20], paying particular attention to whether studies relied on mature clinical trial data or extrapolated immature endpoints, how outcomes were modeled beyond the observed follow‐up, and whether funding and conflicts of interest were disclosed. No numerical scoring system was applied; instead, limitations affecting interpretability and credibility were qualitatively summarized.

### 2.6 Synthesis and Presentation of Cross‐Study Uncertainty

**FIGURE 2:**
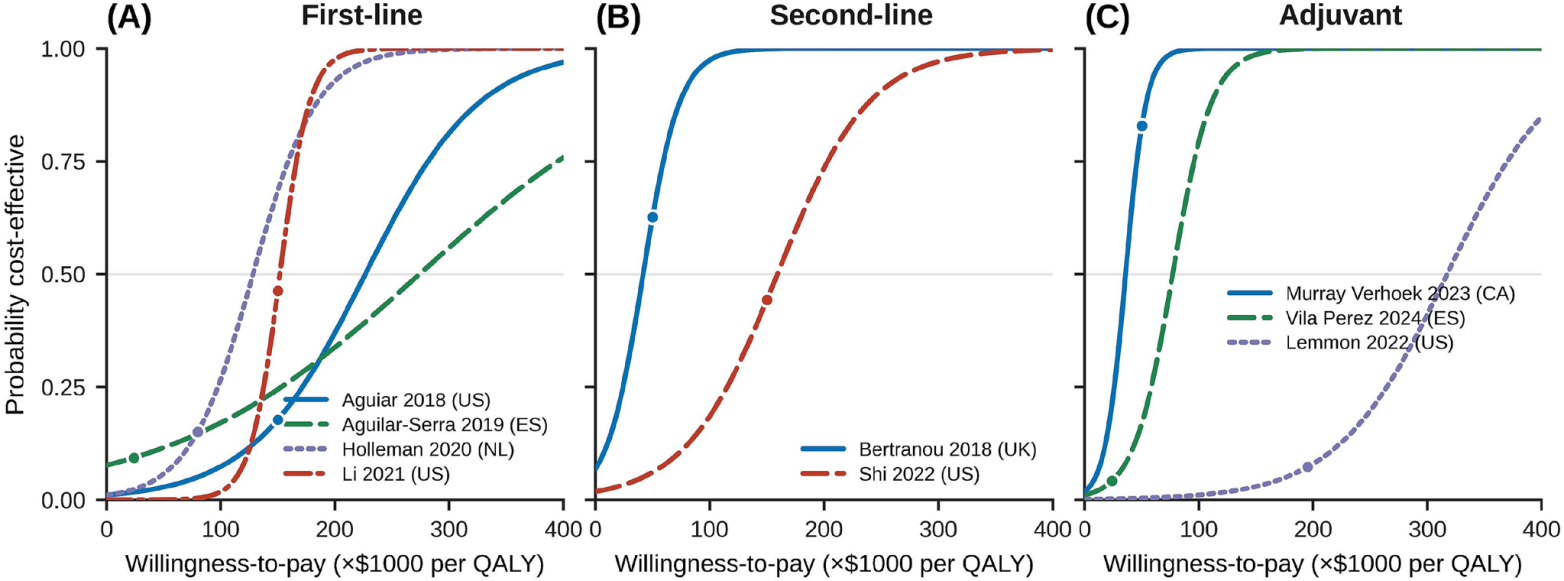
Cost‐effectiveness acceptability curves for osimertinib across treatment settings. Cost‐effectiveness acceptability curves for osimertinib across treatment settings. Each curve gives the modeled probability that osimertinib is cost‐effective as a function of the willingness‐to‐pay threshold, shown on a common scale of US$1000 per QALY to allow visual comparison, for the first‐line (A) [11, 13, 14, 18], second‐line (B) [17, 21], and adjuvant (C) [15, 22, 23] settings; studies are identified in the panel legends and distinguished by line color and style. The filled marker on each curve indicates the probability of cost‐effectiveness at that study's own local willingness‐to‐pay threshold, and the light horizontal line marks a probability of 0.5. The curves are illustrative reconstructions derived from the incremental cost‐effectiveness ratios reported in the included studies and are not reproductions of the original probabilistic output.

**FIGURE 3:**
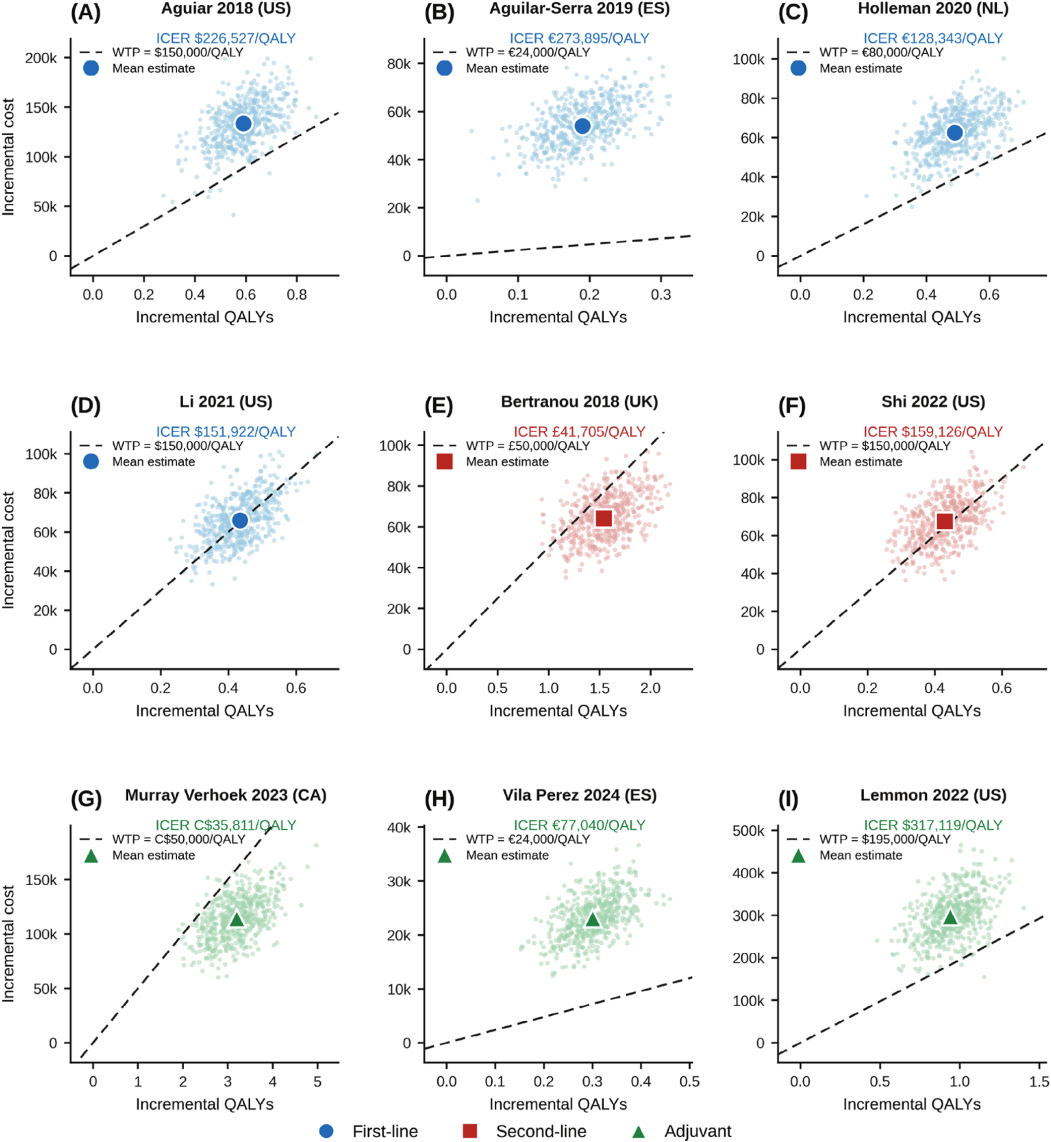
Probabilistic sensitivity analysis across the nine included economic evaluations. Probabilistic sensitivity analysis across the nine included economic evaluations. Each panel (A‐I) [11, 14, 15, 16, 17, 19, 22, 23, 24] shows the cost‐effectiveness plane for one study, with incremental quality‐adjusted life‐years on the horizontal axis and incremental cost in the original currency of that study on the vertical axis (axis values abbreviated, with k denoting thousands). The large marker is the published base‐case estimate and the dashed line is the local willingness‐to‐pay threshold, drawn through the origin with a slope equal to that threshold; a study therefore lies below the line when its ICER is under the local threshold (cost‐effective) and above the line when it exceeds it. The surrounding points are an illustrative reconstruction of the joint uncertainty around the mean and do not reproduce the original Monte Carlo iterations, which were not available in the source publications. In the panel D [18], the plane reflects upfront osimertinib versus a first‐generation TKI (ICER $151,922/QALY); in the same study a sequential strategy was alternatively more effective and less costly. The panel I [23] reflects the corrected published base case (0.94 incremental QALYs; incremental cost $297,760; ICER $317,119 per QALY). Marker color and shape denote treatment setting (blue circle, first‐line; red square, second‐line; green triangle, adjuvant).

Given the heterogeneity described above, a quantitative meta‐analysis was not appropriate. Findings were synthesized narratively within a structured comparative framework, grouping studies by treatment setting, perspective, and region. To help readers visualize cross‐study patterns, Figure 2 presents cost‐effectiveness acceptability curves (CEACs) by treatment setting and Figure 3 presents the cost‐effectiveness plane for the included evaluations, one panel per study. These figures are illustrative reconstructions built from each study's reported base‐case ICERs, incremental costs, and incremental QALYs (Tables 1, 2, 3): in Figure 2 each curve is anchored to cross a probability of 0.5 at the study's reported ICER, and in Figure 3 each study's reported base‐case point is plotted against its local willingness‐to‐pay threshold. They are not reproductions of the original published figures. Because probabilistic‐sensitivity‐analysis iterations were not available from the source publications, Figure 3 displays base‐case point estimates rather than simulated scatter. Because thresholds and currencies differ by jurisdiction, the CEAC abscissa is expressed in each study's native currency and the curves are not directly superimposable; this is stated in the figure legends and considered in interpretation.

## 3 Results

### 3.1 Study Selection and Characteristics

The search identified 1326 records across databases (PubMed/MEDLINE, *n* = 44; Embase, *n* = 762; Web of Science Core Collection, *n* = 501; Cochrane Library, *n* = 19). After removal of 153 duplicates, 1173 records were screened and 1148 were excluded at title/abstract level. Twenty‐five reports were retrieved for full‐text assessment, of which 16 were excluded with documented reasons, leaving 9 studies for qualitative synthesis (Figure 1) [11, 14, 15, 16, 17, 19, 22, 23, 24]. These included four first‐line cost‐effectiveness analyses, two second‐line/sequential evaluations, and three evaluations in the adjuvant setting, all published between 2018 and 2024. Their characteristics, model structures, perspectives, time horizons, discount rates, comparators, and base‐case outcomes are summarized in Tables 1, 2, 3 (first‐line, second‐line, and adjuvant settings, respectively). All nine studies used model‐based cost‐utility analyses, most commonly partitioned‐survival or Markov models, to simulate the course of NSCLC. The analytic perspective was predominantly that of national healthcare payers (United States, United Kingdom, Spain, Canada), and one Dutch evaluation additionally adopted a societal perspective that captured indirect costs such as productivity losses [14]. Time horizons ranged from 8 to 10 years to lifetime, and discount rates were typically 3% to 3.5% for both costs and effects, except for the Dutch model, which applied 4% for costs and 1.5% for effects. Comparators reflected the standard of care at the time each model was developed: earlier‐generation EGFR TKIs in the first‐line setting; platinum‐pemetrexed chemotherapy after TKI failure in the second‐line/T790M‐positive setting; and placebo or routine surveillance after resection (with allowance for adjuvant chemotherapy) in the adjuvant setting.

Figures 2 and 3 summarize the uncertainty around these estimates. Figure 2 presents the reconstructed CEACs by setting, showing the modeled probability that osimertinib is cost‐effective across a range of WTP thresholds; for most first‐line and adjuvant analyses this probability remained low until thresholds well above those actually applied in the corresponding jurisdictions. Figure 3 displays the cost‐effectiveness‐plane scatter derived from the reported probabilistic estimates: most studies cluster in the north‐east quadrant (more effective, more costly), with the notable exception of the US sequential analysis [18], whose cloud lies slightly to the left of the vertical axis, consistent with the small negative incremental QALY reported for upfront osimertinib versus the sequential strategy. As explained in Section 2.6, both figures are schematic reconstructions intended to convey heterogeneity, not re‐analyses of source data.

### 3.2 Clinical Inputs

All models derived efficacy data from the relevant clinical trials (FLAURA for first‐line treatment, AURA3 or similar trials for second‐line therapy in T790M‐positive disease, and ADAURA for adjuvant therapy) or from meta‐analyses of EGFR‐TKI studies [7, 8, 9, 25, 26, 27, 28]. PFS and OS benefits were incorporated through hazard ratios or fitted survival curves. Because all models used long or lifetime horizons, OS had to be extrapolated beyond the observed trial follow‐up; this was particularly relevant in the adjuvant setting, where survival data were immature when the models were built. Extrapolation generally relied on parametric survival models or trial‐based hazard ratios. The toxicity profile of osimertinib was generally favorable; however, the costs and disutility associated with adverse events, including grade ≥ 3 diarrhea, rash, and transaminase elevation, were included when clinically meaningful.

### 3.3 Costs

The acquisition cost of osimertinib, approximately $17,000 per month in the US, was the principal cost driver in every analysis [11, 12]. Comparator costs reflected generic prices for first‐generation TKIs, where available, and local estimates for chemotherapy. Downstream costs, including routine imaging, clinical visits, management of disease progression, and end‐of‐life care, were incorporated to varying degrees. Several evaluations also modeled the cost of subsequent therapies, such as second‐line osimertinib in comparator arms or chemotherapy after progression, particularly in analyses of sequential treatment strategies.

### 3.4 Summary of Economic Outcomes

Incremental QALY gains with osimertinib ranged from 0.19 to 0.59 in first‐line models and from 0.30 to 3.20 in selected second‐line and adjuvant analyses. Incremental costs were consistently high, tens to hundreds of thousands of dollars or euros, resulting in base‐case ICERs that exceeded commonly applied WTP thresholds. In first‐line use, osimertinib provided 0.19–0.59 additional QALYs, yielding ICERs of $151,922–$231,123/QALY in the United States and €128,343–€273,895/QALY in Europe, against thresholds of $100,000–$150,000 and €24,000–€80,000, respectively [11, 13, 14]. One US analysis suggested that sequential treatment, a first‐generation TKI followed by osimertinib at T790M‐positive progression may, under certain assumptions, be more effective and less costly than upfront osimertinib, although the results were sensitive to model inputs [18, 24].

### 3.5 First‐Line Setting: Upfront Osimertinib

Four studies evaluated osimertinib as first‐line therapy for advanced EGFR‐mutated NSCLC [11, 15, 16, 25]. All relied on FLAURA efficacy data and found that upfront osimertinib improved modeled outcomes but at substantially higher costs, resulting in ICERs above the thresholds applied in each setting (Table 1).

**TABLE 1:** Cost‐effectiveness evaluations of first‐line osimertinib in EGFR‐mutated NSCLC.

Author, year, country	Population/setting	Intervention	Comparator	Model type	Perspective/time horizon	Discount rate	Threshold (WTP)	QALY gain	Incremental cost (native currency)	ICER (per QALY, native currency)	Sensitivity analysis	Funding/sponsor	Main conclusion
Aguiar et al., 2018, USA/Brazil [11]	Advanced EGFR‐mutated NSCLC, first‐line	Osimertinib	Erlotinib, gefitinib, afatinib	Partitioned survival	US payer; 10‐year	3%	$100–150 k	0.59	$133,472	$226,527	Deterministic; OS scenarios	Not reported	Not cost‐effective at list price; > 50% price cut needed
Aguilar‐Serra et al., 2019, Spain [13]	Advanced EGFR‐mutated NSCLC, first‐line	Osimertinib	Erlotinib, gefitinib	Markov	Spanish NHS; lifetime	3%	€24,000	0.19	€54,050	€273,895	Probabilistic	Independent	Not cost‐effective; > 60% price cut required
Holleman et al., 2020, Netherlands [14]	Advanced EGFR‐mutated NSCLC, first‐line	Osimertinib	Afatinib, erlotinib, gefitinib	Markov	Societal; lifetime	4% costs; 1.5% effects	€80,000 (end‐of‐life)	0.49	€62,579	€128,343	Probabilistic	Not reported	Not cost‐effective; ≈30% price cut required
Li et al., 2021, USA/China [18]	Advanced EGFR‐mutated NSCLC, first‐line/sequential	Upfront osimertinib	Gefitinib → osimertinib (sequential)	Markov	US payer & Chinese NHS; lifetime	3%	$100–150 k (US); local (CN)	0.43 (US)	$66,000 (US)	$151,922	Probabilistic	Independent	Upfront ICER above threshold; a sequential strategy was alternatively more effective and less costly in the US; both cost‐effective in China after price cuts

*Note:* This table synthesizes model‐based analyses comparing first‐line osimertinib with earlier‐generation EGFR TKIs (or with a sequential strategy). It reports the clinical setting, model structure, perspective, and WTP threshold applied in each jurisdiction, together with incremental QALYs, incremental costs, and ICERs. Across settings, base‐case ICERs exceeded the locally applied thresholds, underscoring the central role of drug pricing in determining value for money. All costs and ICERs are reported in the native currency of each publication ($, US dollars; €, euros; £, pounds sterling; C$, Canadian dollars). No currency conversion, inflation adjustment, or purchasing‐power‐parity adjustment was applied; each ICER should be interpreted against the WTP threshold of its own jurisdiction (see Methods).

Abbreviations: ICER, incremental cost‐effectiveness ratio; NHS, National Health Service; NICE, National Institute for Health and Care Excellence; OS, overall survival; QALY, quality‐adjusted life‐year; TKI, tyrosine kinase inhibitor; WTP, willingness‐to‐pay.

Aguiar et al. (2018, United States and Brazil) used patient‐level FLAURA data and reported a mean gain of 0.59 QALY for first‐line osimertinib compared with first‐ or second‐generation TKIs [11]. From a U.S. payer perspective, ICERs were $226,527/QALY versus erlotinib, and $219,874‐$231,123/QALY versus gefitinib or afatinib, values above conventional U.S. thresholds. In Brazil, lower drug prices reduced ICERs to $162,329–$180,804/QALY, still above the local threshold. ICERs were most sensitive to OS assumptions: they fell to $84,342/QALY under optimistic survival assumptions and rose to $859,771/QALY under pessimistic ones. The authors concluded that a price reduction of more than 50% would be required for first‐line osimertinib to be cost‐effective at 2018 prices.

Aguilar‐Serra et al. (2019, Spain) employed a Markov model and found only a 0.19‐QALY advantage for osimertinib over erlotinib or gefitinib, reflecting a modest PFS gain [13]. Total costs were €83,259 versus €29,209, giving an incremental cost of €54,050 and an ICER of €273,895/QALY, nearly eleven times Spain's threshold of about €24,000/QALY. The probability of cost‐effectiveness at that threshold was below 5%, and the authors estimated that a price reduction exceeding 60% would be needed.

Holleman et al. (2020, Netherlands) conducted a societal‐perspective analysis in which osimertinib yielded the highest QALYs (2.01 vs. 1.36–1.52 for gefitinib, erlotinib, or afatinib) [14]. The incremental cost versus afatinib was €62,579 for a 0.49‐QALY gain, resulting in an ICER of €128,343/QALY, above the Dutch end‐of‐life threshold of €80,000/QALY. Probabilistic analysis indicated that afatinib was more likely to be cost‐effective under Dutch criteria, and a price reduction of at least 30% would be required to bring osimertinib below €80,000/QALY.

Li et al. (2021, United States and China) compared upfront osimertinib with a sequential strategy of first‐line gefitinib followed by osimertinib at T790M progression [18]. In the US analysis, upfront osimertinib had an ICER of $151,922/QALY versus a first‐generation TKI, marginally above the $150,000 threshold; in addition, the sequential strategy (a first‐generation TKI followed by osimertinib at T790M progression) was slightly more effective (0.05 QALYs gained) and less costly than upfront treatment. This result reflected both the high price of osimertinib and the fact that not all patients require it, because not all acquire T790M; it was therefore contingent on the assumed rate of T790M resistance and on the efficacy of second‐line osimertinib, and should be read as scenario‐dependent rather than definitive. In China, by contrast, both strategies were cost‐effective at local thresholds because of substantially lower negotiated prices.

Taken together, these first‐line analyses consistently reported ICERs above the thresholds applied in their respective Western jurisdictions; the modeled QALY gains, generally well under 1.0, were too small to offset the large incremental costs. Approaching cost‐effectiveness typically required price reductions in the order of 20%–60% or substantially higher thresholds. More favorable ICERs emerged only under optimistic survival scenarios, such as the assumption that a proportion of patients are cured, which markedly lowered ICERs but remains unproven [26]. Consistent with this pattern, the reconstructed first‐line CEAC (Figure 2a) shows that the probability of cost‐effectiveness for osimertinib remained below 50% in most analyses even at thresholds approaching $200,000/QALY.

### 3.6 Second‐Line/Sequential Setting: T790M‐Positive Disease

Two studies evaluated second‐line osimertinib in T790M‐positive NSCLC after progression on a first‐ or second‐generation TKI, a scenario that was common before osimertinib moved into first‐line use (Table 2) [17, 21].

**TABLE 2:** Cost‐effectiveness evaluations of osimertinib as second‐line therapy in T790M‐positive NSCLC.

Author, year, country	Population/setting	Intervention	Comparator	Model type	Perspective/time horizon	Discount rate	Threshold (WTP)	QALY gain	Incremental cost (native currency)	ICER (per QALY, native currency)	Sensitivity analysis	Funding/sponsor	Main conclusion
Bertranou et al., 2018, UK [21]	T790M‐positive NSCLC after TKI failure	Osimertinib	Platinum‐pemetrexed chemotherapy	Partitioned survival	NHS; lifetime	3.5%	£30 k (≤ £50 k end‐of‐life)	1.54	£64,283	£41,705	Probabilistic	NICE submission (AstraZeneca)	Acceptable under end‐of‐life criteria; reimbursed with discount
Shi et al., 2022, USA/China [17]	T790M‐positive NSCLC after TKI failure	Osimertinib	Platinum‐pemetrexed chemotherapy	Markov	US payer & Chinese NHS; lifetime	3%	$100–150 k (US); local (CN)	0.43	$67,588 (US)	$159,126 (US)	One‐way; probabilistic	Independent	Borderline in US (37% at $150 k); dominant in China after price cuts

*Note:* This table summarizes evaluations of osimertinib after progression on an earlier‐generation TKI in patients with the T790M resistance mutation. Compared with the first‐line setting, ICERs were closer to (or, in China, below) accepted thresholds, reflecting the poor outcomes of the chemotherapy comparator and the influence of local pricing. All costs and ICERs are reported in the native currency of each publication ($, US dollars; €, euros; £, pounds sterling; C$, Canadian dollars). No currency conversion, inflation adjustment, or purchasing‐power‐parity adjustment was applied; each ICER should be interpreted against the WTP threshold of its own jurisdiction (see Methods).

Abbreviations: ICER, incremental cost‐effectiveness ratio; NHS, National Health Service; NICE, National Institute for Health and Care Excellence; OS, overall survival; QALY, quality‐adjusted life‐year; TKI, tyrosine kinase inhibitor; WTP, willingness‐to‐pay.

Bertranou et al. (2018, United Kingdom) analyzed the model submitted to NICE for the T790M‐positive indication [21]. Drawing on the AURA extension/AURA2 trials for osimertinib and the IMPRESS trial for chemotherapy, the model estimated an additional 1.54 QALYs for second‐line osimertinib versus platinum‐pemetrexed, at an incremental cost of £64,283 and an ICER of £41,705/QALY. Although this exceeded NICE's standard £30,000/QALY threshold, it was deemed acceptable under end‐of‐life criteria (which allow thresholds up to £50,000/QALY), and probabilistic analysis indicated a 63% probability of cost‐effectiveness at £50,000/QALY. These findings supported a positive reimbursement decision, with osimertinib initially funded through the Cancer Drugs Fund. The relatively favorable result reflects the poor outcomes of the comparator (chemotherapy after TKI failure), which amplifies the incremental benefit of osimertinib.

Shi et al. (2022, United States and China) compared second‐line osimertinib with platinum‐based chemotherapy, incorporating AURA3 OS data that did not demonstrate a statistically significant OS benefit (HR 0.87) [17]. From a U.S. payer perspective, osimertinib yielded a gain of 0.43 QALYs at an incremental cost of $67,588, producing an ICER of $159,126/QALY. This slightly exceeded the $150,000 threshold, with a 37% probability of cost‐effectiveness at that threshold. In patients with central nervous system (CNS) metastases, the result was less favorable because of shorter survival in both arms, leading to comparable QALYs but higher costs for osimertinib. In China, negotiated price reductions again rendered second‐line osimertinib dominant. The reported list‐price differential was striking, $0.36 per mg in China versus $6.62 per mg in the United States, and sensitivity analyses confirmed that drug price and OS assumptions were the key US drivers.

Overall, second‐line osimertinib produced ICERs near or slightly above conventional thresholds in Western systems ($159,126/QALY in the United States and £41,705/QALY in the United Kingdom), with probabilistic estimates of cost‐effectiveness of 37% at $150,000/QALY in the US model and more than 60% at £50,000/QALY in the UK model (Figure 2b). NICE approved osimertinib under end‐of‐life criteria, and several payers adopted risk‐sharing or discount agreements [27]. The value proposition was therefore more favorable in the second‐line than in the first‐line setting, but it still sat at the margin of acceptability in the United States in the absence of price concessions, and drug price and survival assumptions remained the principal determinants.

### 3.7 Adjuvant Setting: Early‐Stage Disease

Three studies assessed adjuvant osimertinib in resected stage IB‐IIIA EGFR‐mutated NSCLC, all based on the ADAURA trial, which demonstrated a marked improvement in disease‐free survival (DFS) with 3 years of osimertinib (HR 0.23) (Table 3) [28, 29, 30]. Because OS data were immature when these models were built, each had to make assumptions about long‐term outcomes, in particular, whether and to what extent the DFS benefit would translate into durable OS gains or cure, and the divergence in these assumptions explains most of the heterogeneity in their results. Verhoek et al. (2023, Canada) developed a state‐transition model incorporating a cure fraction, defined as patients remaining disease‐free for at least 5 years [22]. Using Canadian costs, adjuvant osimertinib provided an additional 3.20 QALYs at an incremental cost of C$114,513, corresponding to an ICER of C$35,811/QALY, below the commonly cited Canadian threshold of C$50,000/QALY. The model projected a 10‐year OS of 62.5% with osimertinib versus 39.3% with observation. Scenario analyses demonstrated that results were highly sensitive to the cure‐fraction assumption; in the absence of long‐term cure, the ICER deteriorated substantially. Under its optimistic survival projections, however, the base‐case analysis supported adjuvant osimertinib as cost‐effective in Canada.

**TABLE 3:** Cost‐effectiveness evaluations of adjuvant osimertinib in resected EGFR‐mutated NSCLC.

Author, year, country	Population/setting	Intervention	Comparator	Model type	Perspective/time horizon	Discount rate	Threshold (WTP)	QALY gain	Incremental cost (native currency)	ICER (per QALY, native currency)	Sensitivity analysis	Funding/sponsor	Main conclusion
Verhoek et al., 2023, Canada [22]	Stage IB‐IIIA resected EGFR+ NSCLC (ADAURA)	Osimertinib	Observation	State‐transition with cure fraction	Canadian payer; lifetime	1.5%	C$50,000	3.20	C$114,513	C$35,811	One‐way; probabilistic	AstraZeneca	Cost‐effective under cure assumptions
Vila Pérez et al., 2024, Spain [15]	Stage IB‐IIIA resected EGFR+ NSCLC (ADAURA)	Osimertinib	Observation ± chemotherapy	Partitioned survival	Spanish NHS; 8‐year	3.5%	€24,000	0.30	€23,113	€77,040	Cure‐fraction and price‐cut scenarios	Independent	Not cost‐effective; price reduction required
Lemmon et al., 2022, USA [23]	Stage IB‐IIIA resected EGFR+ NSCLC (ADAURA)	Osimertinib	Placebo/observation	Markov	US payer; 10‐year	3%	up to $195,000	0.94	$297,760	$317,119	Probabilistic; OS‐gain scenarios	Independent	Not cost‐effective; only with major price cuts or OS benefit

*Note:* This table compiles economic models of adjuvant osimertinib based on the ADAURA trial. Incremental QALYs and ICERs show wide heterogeneity: favorable cost‐effectiveness emerged only under optimistic cure or durable‐benefit assumptions (Canada), whereas analyses assuming delayed recurrence (Spain, USA) reported ICERs well above conventional thresholds. The decisive factor in each case was whether the disease‐free‐survival benefit was modeled as translating into durable overall‐survival gains. All costs and ICERs are reported in the native currency of each publication ($, US dollars; €, euros; £, pounds sterling; C$, Canadian dollars). No currency conversion, inflation adjustment, or purchasing‐power‐parity adjustment was applied; each ICER should be interpreted against the WTP threshold of its own jurisdiction (see Methods). Note: all three adjuvant models predated the final ADAURA overall‐survival analysis (Tsuboi et al., 2023 [25]; OS HR 0.49, 5‐year OS 88% vs. 78% in stage IB‐IIIA), which postdates these evaluations and is expected to make the more favorable survival assumptions more plausible; formal re‐analysis with the mature OS data is still required.

Abbreviations: ICER, incremental cost‐effectiveness ratio; NHS, National Health Service; NICE, National Institute for Health and Care Excellence; OS, overall survival; QALY, quality‐adjusted life‐year; TKI, tyrosine kinase inhibitor; WTP, willingness‐to‐pay.

Vila Pérez et al. (2024, Spain) adopted a more conservative approach [15]. Their model estimated a QALY gain of only 0.30, reflecting the expectation that many patients are already cured by surgery with or without chemotherapy, which limits the incremental benefit of osimertinib. The incremental cost was €23,113, yielding an ICER of €77,040/QALY, well above Spain's €24,000/QALY threshold. The authors concluded that adjuvant osimertinib would not be cost‐effective unless its price fell substantially or a clear OS benefit emerged, and their budget‐impact analysis highlighted the financial burden of widespread adoption. Sensitivity analyses indicated that only a price reduction of more than 10%, or strong evidence of a cure effect, would bring the ICER within acceptable limits.

Lemmon et al. (2022, United States) modeled 3 years of adjuvant osimertinib and found a gain of 0.94 QALYs at an additional cost of $297,760, giving an ICER of $317,119/QALY [23]. Even at a generous US threshold of $195,000/QALY, the probability of cost‐effectiveness was negligible, and the high monthly cost of osimertinib was the dominant driver. The authors concluded that only very large price reductions (roughly 50% or more) or a definitive OS benefit would make adjuvant osimertinib economically viable. Importantly, this analysis predated the mature OS data.

The picture in the adjuvant setting has since been clarified by the final ADAURA OS analysis, which was not available to most of these models. In the overall stage IB‐IIIA population, adjuvant osimertinib reduced the risk of death by 51% (OS HR 0.49; 95% CI 0.34–0.70; *p* < 0.001), with a 5‐year OS of 88% versus 78% with placebo; the corresponding figures in the stage II‐IIIA population were 85% versus 73% (HR 0.49; 95% CI 0.33–0.73; *p* < 0.001) [25]. These data strengthen the case for the durable‐benefit and partial‐cure assumptions used in the more favorable models (such as the Canadian analysis) and weaken the purely “delayed‐recurrence” framing adopted by the less favorable ones. Two caveats temper this interpretation, however. First, the distinction between a DFS benefit, a durable OS benefit, and true cure is not merely semantic: only genuine cure, or a sustained divergence of survival curves, generates the large QALY gains needed to offset 3 years of high‐cost therapy, whereas a benefit that merely postpones recurrence yields far smaller gains—precisely the divergence that separates the Canadian result from the Spanish and US results. Second, even the final ADAURA OS analysis remains immature in event terms (roughly 18%–21% of expected deaths, with median OS not reached), so meaningful extrapolation uncertainty persists, and formal re‐analysis of the published models with the mature OS data is still required before the favorable Canadian estimate can be considered generalizable. Pending such re‐analysis, several systems have taken a cautious stance; NICE, for example, has provided access to adjuvant osimertinib through managed‐access arrangements rather than routine commissioning. In summary, adjuvant cost‐effectiveness was the most variable of the three settings (Table 3). Incremental QALYs ranged from 0.30 (Spain) to 3.20 (Canada), and ICERs from C$35,811/QALY under cure assumptions to $317,119/QALY under delayed‐recurrence assumptions. The decisive factor in every case was whether the DFS benefit was modeled as translating into durable OS gains. The mature ADAURA OS data make the more optimistic survival assumptions more plausible than they were at the time of the original analyses, but they do not by themselves establish cost‐effectiveness at current prices, and updated modeling is needed.

### 3.8 Quality of Evidence

Reporting quality, appraised with CHEERS 2022, was generally adequate. Most studies described their model structure, perspective, time horizon, and funding, and most included sensitivity analyses, though the rigor and scope of those analyses varied. Holleman et al. (2020) [14] and Verhoek et al. (2023) [22] conducted full PSAs with CEACs, whereas Aguiar et al. (2018) [11] and Shi et al. (2022) [17] relied chiefly on deterministic sensitivity analyses. Justification of comparator choice and survival modeling was detailed in some studies (e.g., Holleman et al. and Shi et al.) but limited in others, and disclosure of funding and conflicts of interest was inconsistent, explicit in some industry‐funded analyses but absent in others. Among the included studies, the UK model by Bertranou et al. [21] and the US models by Aguiar et al. [11] and Shi et al. [17] were of comparatively higher reporting quality, reflecting their use of mature randomized data (FLAURA or AURA3), clearly stated assumptions, and transparent reporting. Several adjuvant analyses, by contrast, depended on strong assumptions about cure and extrapolated survival, which introduced substantial uncertainty [31]. The robvis assessment indicated that all studies raised at least some concerns in one or more domains. Time‐horizon considerations were relevant for models using fixed 10‐year horizons [22], which may understate long‐term benefit, and for studies requiring extensive lifetime extrapolation. Structural concerns arose where sequential EGFR‐TKI use was modeled in one comparator arm but not another [14]. Approaches to parameter uncertainty were inconsistent: full PSAs were performed in some studies [11, 19] but not others [11, 26], limiting insight into joint uncertainty. Only a subset of studies reported full PSAs with CEACs [11, 14, 23]; in the reconstructed cost‐effectiveness plane (Figure 3), most iterations fall in the north‐east quadrant, consistent with base‐case ICERs above commonly accepted thresholds. Within this limited set, osimertinib was, at its full list price, predominantly not cost‐effective.

## 4 Discussion

This review brings together the published economic evaluations of osimertinib across first‐line, second‐line, and adjuvant settings in EGFR‐mutated NSCLC. Despite clear clinical benefits, including improvements in PFS and OS, enhanced CNS control, and a favorable toxicity profile, osimertinib frequently exceeded the WTP thresholds applied in Western health systems ($100,000–$150,000/QALY in the United States and €24,000–80,000/QALY in Europe) [14, 15, 19, 25]. A prior systematic review by Nargesi et al. reported similarly high ICERs [32]; our review extends that work by incorporating more recent adjuvant analyses and the now‐mature ADAURA OS data, and by interpreting each estimate against its own local threshold. We emphasize at the outset that these conclusions derive from only nine heterogeneous modeling studies and should be read as indicative rather than definitive. At prevailing list prices, upfront first‐line osimertinib was generally not cost‐effective. Aguiar et al. estimated $226,527/QALY in the United States [11], Aguilar‐Serra et al. €273,895/QALY in Spain (close to an order of magnitude above the local threshold) [13], and Holleman et al. concluded that osimertinib was not cost‐effective at the Dutch €80,000/QALY threshold [14]. Li et al. demonstrated that beginning with a lower‐cost first‐generation TKI and reserving osimertinib for T790M‐positive progression could offer better economic value than treating all patients upfront [18]. Although clinical benefits are well documented, the median PFS advantage of approximately 8–9 months observed in the FLAURA trial has not, by itself, been sufficient to meet cost‐effectiveness benchmarks at current prices in Western healthcare systems [8, 9].

The second‐line/T790M‐positive setting was more favorable, largely because the comparator, chemotherapy after TKI failure, performs poorly, which widens the incremental benefit of osimertinib. The UK NICE appraisal accepted an ICER of £41,705/QALY under end‐of‐life criteria, and reimbursement followed with a confidential discount [21, 27]. Shi et al. estimated a U.S. ICER of $159,126/QALY, only marginally above the level some U.S. payers might tolerate [17]. Even here, however, the US ICER sat at the margin of acceptability, and probabilistic analysis put the chance of cost‐effectiveness below 50% at a $150,000/QALY without price concessions. The contrast across jurisdictions shows that the economic value of osimertinib is not intrinsic but depends heavily on price and the local threshold.

The adjuvant setting generated the widest variation, and it is here that the evidence has moved most since the original models were published. A Canadian model assuming a cure fraction reported osimertinib as cost‐effective (C$35,811/QALY) [22], whereas a U.S. analysis ($317,119/QALY) [23], and a Spanish analysis (€ 77,040/QALY) [15] placed it well above accepted thresholds. The difference turns almost entirely on long‐term survival assumptions: if osimertinib cures a meaningful fraction of patients, the QALY gains are large and the drug can look cost‐effective; if it merely delays recurrence without changing ultimate outcomes, the gains are small and the ICER is high. The interim ADAURA data had already shown a striking DFS benefit (89% of osimertinib‐treated patients alive and disease‐free at 24 months versus 52% with placebo) [29], and the final OS analysis now demonstrates a significant OS benefit (HR 0.49; 5‐year OS 88% vs. 78% in stage IB‐IIIA) [25].

This shifts the balance of plausibility towards the durable‐benefit assumptions, but, with OS maturity still low and median OS not reached, it does not settle the question; the published cost‐effectiveness models need to be re‐run with the mature data before firm conclusions can be drawn. Until then, adjuvant osimertinib remains an economically high‐stakes choice: a three‐year course approaches half a million US dollars per patient, and several systems, including NHS England, through a managed‐access scheme, have granted access conditionally pending definitive survival evidence [27, 33, 34, 35].

The economics of combination strategies are even less favorable. Adding chemotherapy to adjuvant osimertinib produced only marginal additional benefit at much higher cost, with an ICER of $380,348/QALY versus $213,448/QALY for osimertinib monotherapy [31]. This finding suggests that osimertinib monotherapy is more economically favorable than combination therapy in the adjuvant setting, although even monotherapy remains not cost‐effective under conventional thresholds. In the metastatic setting, early analyses of first‐line osimertinib plus chemotherapy (FLAURA2) likewise reported ICERs above $200,000/QALY with a low probability of cost‐effectiveness at $150,000/QALY [36], reinforcing the conclusion that more intensive, more expensive regimens worsen rather than improve value at current prices.

Cross‐country comparisons underline how decisively price and thresholds shape these results. In Spain, with a threshold near €24,000/QALY, osimertinib was not cost‐effective in any setting [15]. Canada, with a threshold around $50,000/QALY, found second‐line use acceptable under end‐of‐life criteria and adjuvant use cost‐effective only under cure assumptions [22, 37]. In the United States, with implicit thresholds often exceeding $150,000/QALY, ICERs of $151,922–$317,119/QALY still left osimertinib outside acceptable limits in most models [36]. Where prices were deeply discounted through national negotiation, as in China, osimertinib became cost‐effective or even cost‐saving [33]. Reimbursement decisions have therefore varied widely across jurisdictions, tracking local pricing and threshold conventions rather than any fixed property of the drug.

A recurring theme is the maturity of survival data. Early first‐line models, based on immature OS data, projected unfavorable ICERs; subsequent FLAURA updates confirmed an OS benefit but did not materially change the cost‐effectiveness profile at list price [9, 23]. In the adjuvant setting, the DFS benefit is well established, and the OS benefit is now demonstrated, but its long‐term magnitude, and hence its economic value, still depends on extrapolation. Models built on optimistic survival assumptions can produce favorable ICERs, whereas more conservative assumptions push results well above conventional thresholds [31]. This sensitivity to evidence maturity is a strong argument for conditional or outcome‐based reimbursement while longer‐term data accrue. This analysis suggests several implications for decision‐makers. Firstly, it is evident that drug price negotiation represents the most direct and impactful lever for improving osimertinib's cost‐effectiveness. Many studies suggest that reductions of 20 to 60% would be required in Western settings [23, 34]. Such reductions have already been achieved in China and in certain regions of Europe through national or confidential agreements. Secondly, managed‐access and risk‐sharing schemes, such as those used by NICE, allow provisional coverage while evidence matures. Thirdly, treatment sequencing deserves consideration. For some patients, starting with a less expensive TKI and reserving osimertinib for progression may offer better value [18, 35], which raises the policy question of whether payers should encourage sequencing on economic grounds even when clinicians prefer upfront osimertinib for its efficacy and CNS activity. Targeting osimertinib to the patients who stand to gain most, for example, those with CNS involvement or those intolerant of earlier‐generation TKIs, may also improve average value, although such selection must be weighed against clinical equity and the practical difficulty of identifying these groups prospectively. From a policy standpoint, the consistently high ICERs argue for outcome‐based pricing. Managed‐entry agreements, risk‐sharing contracts, and price–volume arrangements, several already in use by payers such as NICE, can align reimbursement with real‐world effectiveness and offer a pragmatic route to interim access while OS data mature. The Six‐Delta outcome‐based contracting framework, for instance, has been applied as a proof of concept to osimertinib in NSCLC, illustrating how price might be tied to performance using real‐world and trial data [38], and performance‐based risk‐sharing agreements have been implemented in other NSCLC and oncology settings, including immunotherapy [39, 40]. Embedding cost‐effectiveness evidence in national guidelines could further support equitable and sustainable access, particularly in publicly funded systems.

Methodologically, manufacturer‐funded studies tended to reach more favorable outcomes than independent evaluations, consistent with prior evidence on industry sponsorship [41]. Although most studies followed CHEERS, transparency varied, and clearer reporting of model structure, assumptions, and sensitivity analyses would strengthen the literature. Future models should integrate real‐world data on recurrence patterns, post‐progression survival, and CNS outcomes, all of which influence osimertinib's value, and should be updated as new evidence, such as the FLAURA2 combination data and the mature ADAURA OS results, becomes available. The long‐term affordability of osimertinib will likely depend on value‐based pricing or outcomes‐based contracts in which payment is linked to survival benefit [42, 43].

This review has several strengths: a pre‐registered protocol, a reproducible multi‐database search with full Boolean strings, duplicate independent screening and extraction, formal CHEERS‐based appraisal, and a structured comparative framework that interprets each ICER against its own local threshold. Nevertheless, important limitations must temper its conclusions. First, eligibility was restricted to North American and European analyses to improve comparability of reimbursement frameworks and thresholds; this geographic restriction introduces a selection bias and limits generalizability to settings, including much of Asia and many low‐ and middle‐income countries, where prices and thresholds differ markedly. The two included dual‐jurisdiction studies illustrate this directly, since their Chinese results (cost‐effective or cost‐saving) diverge sharply from their Western results. Second, we included only peer‐reviewed full publications and excluded conference abstracts, editorials, and gray literature, including unpublished HTA reports. Because pharmacoeconomic findings, and unfavorable ICERs in particular, are often reported in HTA dossiers or conference proceedings rather than journals, this restriction may introduce publication bias and omit relevant analyses; we accepted this trade‐off to ensure that every included study could be appraised in full methodological detail, but we acknowledge that it narrows the evidence base. Third, the number of eligible studies was small (nine) and they were highly heterogeneous in structure, assumptions, comparators, and setting, which precluded meta‐analysis and means that our synthesis is qualitative. Fourth, we did not normalize costs across studies: values are reported in native currencies without adjustment for inflation, exchange rates, or purchasing power parity. We judged that converting jurisdiction‐specific, internally consistent models would distort them, and we therefore compared each ICER with its own local threshold; however, this approach limits direct numerical comparison of absolute ICERs across countries, and readers should bear this in mind. Finally, the CEACs and cost‐effectiveness‐plane scatter in Figures 2 and 3 are illustrative reconstructions built from reported summary statistics, not reproductions of the original analyses, and the CEAC abscissa mixes native currencies; they should be read as schematic aids to visualizing heterogeneity rather than as pooled re‐analyses.

## 5 Conclusions

Across the available Western economic evidence, osimertinib frequently exceeds commonly accepted cost‐effectiveness thresholds at current list prices, particularly in the first‐line and adjuvant settings, while showing more favorable value in the second‐line/T790M‐positive setting under end‐of‐life criteria or confidential pricing. These conclusions rest on a small, heterogeneous body of modeling studies and should be interpreted cautiously. The long‐term affordability and sustainability of osimertinib will likely depend on substantial price reductions, prioritization of patients with the greatest absolute benefit, maturation and formal incorporation of OS data, especially in the adjuvant setting, where the final ADAURA analysis now shows a significant OS benefit, and reimbursement strategies that more closely link payment to real‐world outcomes.

## Author Contributions

**Nicola Perrotta:** investigation, conceptualization, writing – original draft, methodology, software, supervision. **Adriana Coluccia:** data curation, software, formal analysis, writing – original draft. **Giulia Amato:** data curation, formal analysis, visualization. **Giacomo Polito:** supervision, writing – review and editing, validation, investigation. **Francesca Rossi:** data curation, formal analysis, visualization.

## Funding

This research did not receive any specific grant funding from public, commercial, or not‐for‐profit sectors.

## Ethics Statement

Ethics approval was not required, as this study did not involve direct research with human participants.

## Conflicts of Interest

The authors declare no conflicts of interest.

## Supporting information


**Data S1:** Supporting Information.

## Data Availability

The data that support the findings of this study are available from the corresponding author upon reasonable request.
