# Supplementary material for: Pharmacoeconomic Analysis of Osimertinib Compared to Earlier‐Generation EGFR Inhibitors in EGFR‐Mutated NSCLC: A Systematic Review
Source: Cancer Med. 2026 Jul 22;15(7):e72129. doi: 10.1002/cam4.72129 (PMC13390182; doi:10.1002/cam4.72129)
Supplement: Supplementary file 1 — Data S1: Supporting Information. [file CAM4-15-e72129-s001.pdf]

Supplementary appendix

Pharmacoeconomic analysis of Osimertinib compared to earlier-generation EGFR inhibitors in EGFR-mutated NSCLC: a systematic review

Search strategy and information sources

The literature search was designed and conducted in accordance with the Preferred Reporting Items for Systematic Reviews and Meta-Analyses (PRISMA 2020) recommendations.

A comprehensive systematic search was performed to identify published pharmacoeconomic studies evaluating osimertinib in epidermal growth factor receptor (EGFR)-mutated non-small cell lung cancer (NSCLC).

The following electronic databases were searched from database inception through August 31, 2025:

- PubMed/MEDLINE
- Embase
- Web of Science Core Collection
- Cochrane Library

Additional studies were identified through:

- backward reference screening of eligible studies;
- forward citation tracking using Google Scholar.

Searches were restricted to:

- English-language studies;
- human studies;
- peer-reviewed publications.

Conference abstracts, editorials, letters, narrative reviews, preprints, and grey literature sources lacking complete peer-reviewed methodological details were excluded to ensure inclusion of fully evaluable pharmacoeconomic analyses.

PubMed/MEDLINE search strategy

The PubMed/MEDLINE search combined terms related to osimertinib, EGFR-mutated NSCLC, and pharmacoeconomic outcomes. Searches were performed primarily within titles and abstracts.

(osimertinib OR Tagrisso OR “osimertinib mesylate”)

AND

(“non-small cell lung cancer” OR NSCLC OR “lung adenocarcinoma”)

AND

(EGFR OR “epidermal growth factor receptor” OR “EGFR mutation” OR T790M)

AND

(“cost-effectiveness” OR “cost utility” OR “economic evaluation” OR pharmacoeconomics OR ICER OR QALY OR “budget impact”)

Embase search strategy

The Embase search used a combination of controlled vocabulary and free-text terms related to osimertinib, EGFR-mutated NSCLC, and economic outcomes.

(osimertinib OR Tagrisso)

AND

(“non-small cell lung cancer” OR NSCLC OR “lung adenocarcinoma”)

AND

(EGFR OR “EGFR mutation” OR T790M)

AND

(“cost-effectiveness” OR “economic evaluation” OR pharmacoeconomics OR ICER OR QALY OR “budget impact”)

Web of Science Core Collection search strategy

TS=(osimertinib OR Tagrisso)

AND

TS=(“non-small cell lung cancer” OR NSCLC OR “lung adenocarcinoma”)

AND

TS=(EGFR OR “EGFR mutation” OR “epidermal growth factor receptor” OR T790M)

AND

TS=(“cost-effectiveness” OR pharmacoeconomics OR “economic evaluation” OR “cost utility” OR ICER OR QALY OR “budget impact”)

Cochrane Library search strategy

(osimertinib OR Tagrisso)

AND

(“non-small cell lung cancer” OR NSCLC)

AND

(EGFR OR “EGFR mutation” OR T790M)

AND

(“cost-effectiveness” OR pharmacoeconomics OR “economic evaluation” OR ICER OR QALY)

Additional search procedures

To maximize retrieval of relevant studies:

1. The reference lists of all eligible articles were manually screened.
2. Forward citation tracking was performed using Google Scholar.
3. Duplicate records were removed before title and abstract screening.
4. Two independent reviewers screened titles, abstracts, and full texts.
5. Disagreements between reviewers were resolved through discussion and, when necessary, adjudication by a third investigator.

Eligibility criteria

Inclusion criteria

- Adult patients with EGFR-mutated NSCLC;
- Studies evaluating osimertinib in first-line, second-line, sequential, or adjuvant settings;
- Cost-effectiveness, cost-utility, budget-impact, or pharmacoeconomic analyses;
- Comparisons with earlier-generation EGFR-TKIs or chemotherapy;
- Economic evaluations informed by randomized clinical trial data or model-based analyses;
- Studies conducted from payer or healthcare-system perspectives;
- Studies performed within North American or European healthcare systems.

Exclusion criteria

- Non-original articles;
- Conference abstracts;
- Editorials and letters;
- Narrative reviews;
- Preclinical studies;
- Studies lacking economic outcomes;
- Studies not published in English;
- Grey literature sources and unpublished HTA reports lacking complete methodological details.

Data extraction and methodological assessment

Two reviewers independently extracted data using a standardized form. Extracted variables included:

- study design;
- healthcare-system perspective;
- treatment setting;

- comparators;
- model structure;
- time horizon;
- discount rates;
- incremental costs;
- quality-adjusted life years (QALYs);
- incremental cost-effectiveness ratios (ICERs);
- willingness-to-pay thresholds;
- sensitivity analyses.

When available, probabilistic sensitivity analyses, cost-effectiveness acceptability curves, and scenario analyses were also collected.

Methodological quality and reporting transparency were assessed according to the Consolidated Health Economic Evaluation Reporting Standards (CHEERS 2022) checklist.

Approach to evidence synthesis

Because of substantial heterogeneity in model structures, survival extrapolation methods, healthcare-system perspectives, pricing assumptions, and willingness-to-pay thresholds, quantitative pooling or meta-analysis of economic outcomes was not considered methodologically appropriate.

Accordingly, results were synthesized narratively using a structured comparative framework across first-line, second-line/sequential, and adjuvant treatment settings.

Methodological scope and rationale

The review focused specifically on North American and European healthcare systems to improve comparability across reimbursement frameworks, drug pricing structures, and willingness-to-pay thresholds.

This geographic restriction was considered appropriate given the substantial variability in healthcare financing systems and pharmacoeconomic thresholds across regions.
